# Liver transplantation as a treatment for Wilson’s disease with neurological presentation: a systematic literature review

**DOI:** 10.1007/s13760-022-01872-w

**Published:** 2022-01-26

**Authors:** Tomasz Litwin, Jan Bembenek, Agnieszka Antos, Adam Przybyłkowski, Marta Skowrońska, Iwona Kurkowska-Jastrzębska, Anna Członkowska

**Affiliations:** 1 Second Department of Neurology, Institute of Psychiatry and Neurology, Sobieskiego 9, 02-957 Warsaw, Poland; 2 Department of Clinical Neurophysiology, Institute of Psychiatry and Neurology, Warsaw, Poland; 3 Department of Gastroenterology and Internal Medicine, Medical University of Warsaw, Warsaw, Poland

**Keywords:** Wilson’s disease, Liver transplantation, Copper, Neurological symptoms, Systematic review

## Abstract

**Introduction:**

Wilson’s disease (WD) is a potentially treatable, genetic disorder of copper metabolism, with survival similar to healthy populations if controlled. However, in almost 50% of WD patients, neurological symptoms persist despite treatment, and in up to 10% of patients, neurological deterioration is irreversible. International guidelines on WD treatment do not recommend liver transplantation (LT) as a treatment for neurological symptoms in WD. However, such treatment has been assessed in retrospective analyses, case and series reports. We aimed to systematically assess all available evidence on the effectiveness and safety of LT in WD patients with neurological presentation.

**Methods:**

This systematic literature review was performed according to Preferred Reporting Items for Systematic Reviews and Meta-Analyses (PRISMA) guidelines. Studies were identified by searching the PubMed database (up to 6 April 2021) and by screening reference lists.

**Results:**

Based on the systematic literature review, 48 articles were identified, showing outcomes of LT in 302 WD patients with neurological symptoms. Of these patients, major improvement was found in 215 cases (71.2%), with no difference in neurological status before and after LT in 21 cases (6.9%). There were 29 deaths (9.6%), neurological worsening in 24 cases (7.9%), and 13 cases (4.3%) were lost to follow-up.

**Conclusions:**

The results suggest that LT is a promising method of WD management in patients with severe, neurological symptoms, particularly if the patient has not responded to pharmacological de-coppering treatment. Further studies of LT in these patients are warranted.

**Supplementary Information:**

The online version contains supplementary material available at 10.1007/s13760-022-01872-w.

## Introduction

Wilson’s disease (WD) is a genetic disorder characterized by pathological copper accumulation in various organs (mainly liver and brain) with damage to the affected organs and clinical symptoms related to injury (mainly hepatic and/or neurological) [1–4].

WD is caused by mutations in *ATP7B*, a gene located on chromosome 13, which encodes the copper-transporting ATPase, ATP7B, which is abundant in the liver and is also found in the brain, placenta, kidneys, lungs and heart [1–3]. Due to ATP7B dysfunction, copper accumulates in hepatocytes and cannot be incorporated into cuproproteins (e.g., ceruloplasmin) and removed into the systemic circulation [1, 3]. Mitochondria are particularly sensitive to copper overload [1–3]. Copper-induced lipid peroxidation leads to mitochondria membrane damage, subsequent disruption of respiratory chain enzymes, and finally hepatocyte necrosis, with release into the circulation of free non-ceruloplasmin-bound copper (NCC) [1–5].

Treatment of WD is based on drugs that induce negative copper balance: (1) chelators (d-penicillamine or trientine) promote increased urinary copper excretion; (2) zinc salts inhibit copper absorption from the digestive tract; and (3) complexors (molybdenum salts, currently in clinical trials) decrease absorption of copper from the digestive tract, promoting excretion of copper into bile, and form complexes with copper and albumins to reduce NCC levels in the systemic circulation [1–5].

Mortality rates in patients with WD are similar to healthy populations if the disease is diagnosed early and appropriately treated [6–12]. In almost 85% of treated WD patients, clinical improvement is observed [13]. However, in 50% of patients with neurological manifestations, clinical symptoms persist, and irreversible neurological deterioration occurs in almost 10% of patients. These findings contribute to the search for other strategies to treat severe neurological WD presentation, such as symptomatic treatments and also liver transplantation (LT) [3, 6–12, 14, 15].

LT is currently recommended only to WD patients with acute liver failure or decompensated liver cirrhosis (despite anti-copper treatment) [1–5, 15]. LT in neurological presentations of WD is still being debated [15]; however, it seems an attractive method as, in contrast to autoimmune hepatitis, primary sclerosing cholangitis, primary biliary cirrhosis, viral hepatitis or alcohol liver disease, WD will not recur in the transplanted organ [1]. There are several retrospective analyses of registries, series reports, and case reports published on LT in WD (including patients with neurological symptoms) as well as small prospective analyses [16–73]. Here, we describe results from the first-ever systematic review of the literature analyzing the efficacy of LT in the treatment of neurological symptoms of WD.

## Materials and methods

### Search strategy and eligibility criteria

This systematic review was performed in concordance with international accepted criteria of the Preferred Reporting Items for Systematic Reviews and Meta-analyses (PRISMA) statement [74].

We searched the PubMed database (up to 6 April 2021) for original studies (prospective and retrospective), as well as case and series reports analyzing the efficacy of LT as a treatment option for WD patients with neurological phenotypic manifestation. Search terms included (“Wilson’s disease” and “liver transplantation” and “neurological symptoms”), (“Wilson’s disease” and “liver transplantation” and “neurology”), and (“Liver transplantation and neurological Wilson’s disease”). Studies eligible for further analysis were: (1) conducted with humans; (2) original studies (prospective or retrospective); (3) case and series reports of the patients with neurological manifestation of WD who have had LT; and (4) in English language. Review articles and studies describing the neurological outcome of LT in patients with other etiologies of liver failure were excluded. The reference lists of extracted publications were also searched.

All identified studies were analyzed and verified independently by all authors to confirm the inclusion criteria, and were grouped as: (1) prospective studies which aimed to present the efficacy of LT in the treatment of neurological symptoms of WD; (2) retrospective studies presenting data from national or center-based LT registries which presented additionally neurological WD patients and their outcome after LT; (3) series reports; and (4) case reports of LT in neurological WD patients.

## Results

**Fig. 1:**
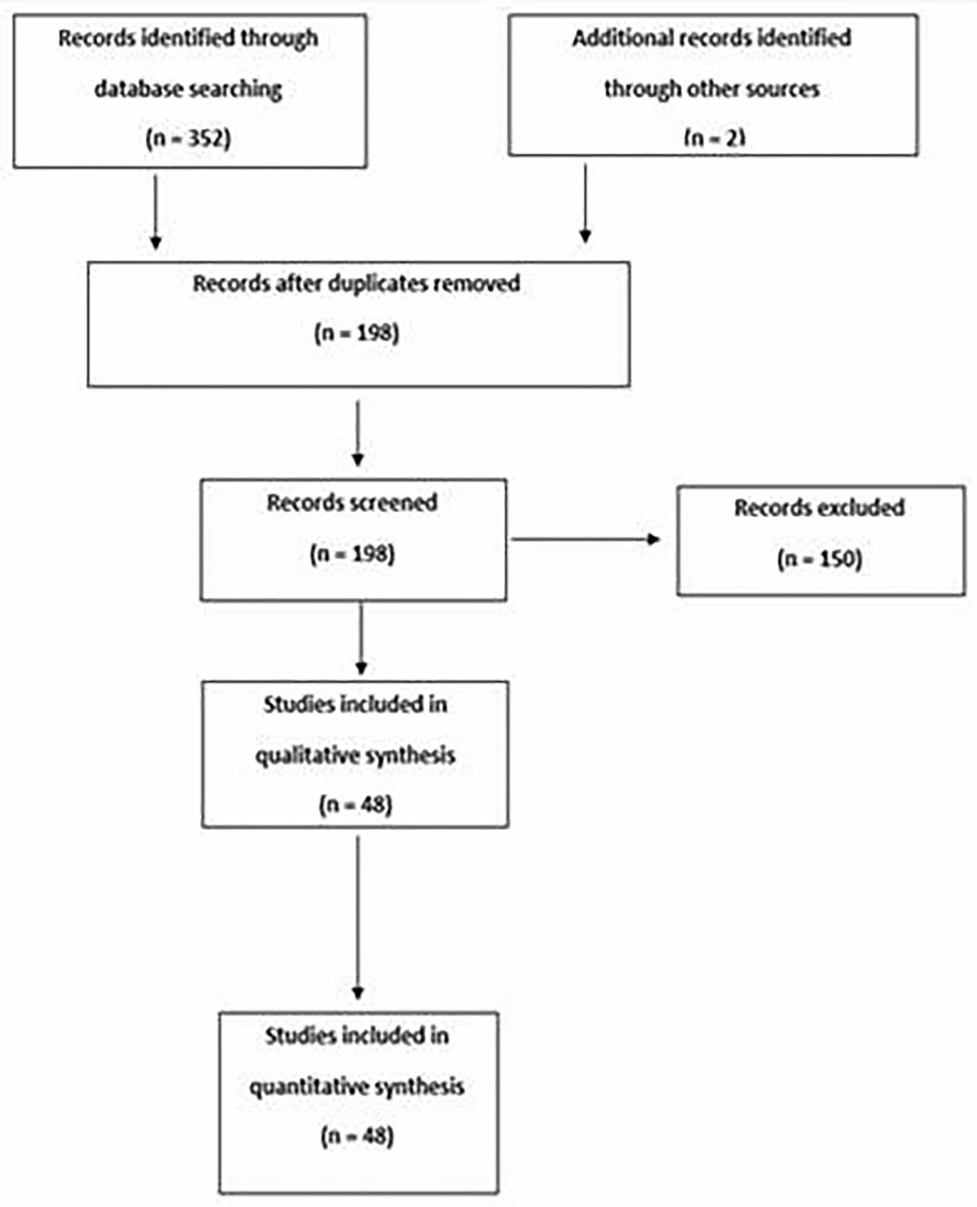
PRISMA search diagram. A total of 352 articles were found during the initial screening and 48 articles were included in the qualitative synthesis

In total, 354 records were retrieved: 352 from PubMed searches and 2 for the detailed review of the reference lists [16, 18] (Fig. 1). Following duplicate removal, 198 publications remained. The title, abstracts and full texts were then screened for relevance, removing another 150 records. Finally, 48 full-text articles of LT in 302 WD patients with neurological symptoms were included in the analysis. No prospective studies were identified, and there were 24 retrospective studies on patients with WD treated with LT; 23 of them were additional analyses of neurological symptoms in WD patients undergoing LT due to liver failure and only 1 was an analysis of neurological symptoms after LT in WD patients transplanted due to neurological deterioration (Table 1). There were 24 case reports and case series describing the WD patients with neurological presentation who had LT (Supplementary Table 1). The methodology for assessing neurological symptoms differed, also the brain magnetic resonance imaging (MRI) data of these patients were of different quality. Moreover, different neurological scales were used, making it impossible to perform meta-analyses of the reviewed studies.

**Table 1:** Summary of studies of WD patients with neurological symptoms who underwent liver transplantation

Authors	Details of the patient population and study	Information on age at LT, time to LT from WD diagnosis, prior treatment and duration of follow-up (if available)	Details of the neurological assessment and MELD score before LT (if available)	Results
Ocal et al. [28]	*N* = 4 (LT indication: chronic liver disease) Retrospective analysis of 53 WD patients	Mean age at LT: 35.2 years (all patients) Treatment type and duration not available	Neurological symptoms description	Tremor decreased in 3/4 WD patients
Poujois et al. [64]	*N* = 18 (LT indication: pure neurological—severe neurological, mRS ≥ 4 and constant neurological deterioration apart WD treatment) Study included 2 patients from Laurencin et al. [35] Retrospective study	Median age at LT: 18.5 years (range 16–20.8) Median time to LT: 6.7 months (range 5.3–14.5) 11 patients initially treated with DPA, 6 on trientine, 1 on trientine and ZS. 12 switched to other WD treatments before LT Median follow-up: 40.9 months (18.1–93.2)	Neurological examination, UWDRS, mRS brain MRI score Median MELD: 8.5 (7–10.8)	Major neurological improvement in 8/18 WD patients, moderate in 4/18, stable in 2/18 Median improvement in UWDRS from 96 (75–112) to 38 (18–56) Improvement in mRS pre-LT 5 vs. 1.5 Improvement in brain MRI score: 7/18 had 75% decrease of MRI score, 7/18 had 33% decrease of MRI score 4/18 died
Ferrarese et al. [27]	*N* = 10 (LT indication: 2 had isolated neuropsychiatric phenotype; 9 had chronic liver disease) Retrospective study of 29 WD patients (27 analyzed)	Mean age at LT: 26 years (range 21–47) Mean time to LT: 7.5 years Mean follow-up: 72 months (range 0.3–130)	Neurological symptoms, UWDRS (6 patients) Mean MELD: 28 (11–49)	No significant difference in 6 patients with available UWDRS before and post-LT Post-LT course did not differ between patients with hepatic and mixed (with neuropsychiatric) presentation
Choudhary et al. [33]	*N* = 3 (LT indication: chronic liver disease) Retrospective study of 18 WD patients	Mean age at LT: 30. 6 years Mean time to LT: 3 years (all patients) Treated with DPA and ZS at time of LT (no detailed data) Mean follow-up: 15 months (range 8–38)	Neurological symptoms description	1 patient fully recovered 2 patients partially recovered
Lankarani et al. [65]	N = 60 (LT indication: chronic liver disease) Retrospective study of 107 WD patients	Range of years at LT: 5–59 Treatment type and duration not available Follow-up: 4015 days	Neurological symptoms description Mean MELD for chronic liver disease group: 20 (range 17.7–24)	Neuropsychiatric manifestation improved in 40 WD patients (67%) Neurological manifestations that were reduced post-LT vs. pre-LT included tremor (18% vs. 41%), ataxia (11% vs. 32%), gait disorder (7% vs. 18%), fine.m.task (3% vs. 18%) and depression (5% vs. 24%) No change in 2 patients Exacerbation of neurological symptoms in 18 patients (mostly drug related)
Yagci et al. [36]	*N* = 9 (LT indication: chronic liver disease) Retrospective study of 42 WD patients	Median age at LT: 19 years (range 10–25) Treatment type and duration not available Follow-up: 36.6 months	12-item neurological scoring system (12–60 pts, where 1 pt is none and 5 pts is severe for each item) 19-item neuropsychological scoring system (0–19 pts, where 1 pt is present) Mean MELD: 18.3 (range 15–26)	Neurological scores improved (mean pre-LT 17.7 vs. post-LT 12.7), with worsening in 1 patient Neuropsychological scores improved (pre-LT 9.0 vs. post-LT 7.0), with progression in 1 patient
Guillaud et al. [19]	*N* = 7 (LT indication: neurological symptoms) *N* = 19 (LT indication: neurological symptoms plus 2 with acute LF and 17 with chronic LF) Retrospective analysis of 128 WD patients	Median age at LT: 22 years (range 7–66) (all patients) Median time from WD diagnosis to LT: 0.3 years (0–30) (all patients) WD treatment not provided for all patients Median follow-up: 72 months	Neurological symptoms description Mean MELD for 7 patients transplanted from neurological reasons: 10 (7–11)	Of the 7 patients transplanted for neurological reasons: 3 patients in major neurological improvement 3 died (2 months, 4 months and 36 months due to infections) Data unavailable for 1 patient Of the 19 patients transplanted for neurological and hepatic reasons 1 died 2 days after LT 3 patients completely recovered 5 partially recovered 1 stabilized 1 had initial worsening of neurological symptoms Data unavailable for 8 patients
Peedikayil et al. [41]	*N* = 4 (LT indication: chronic LF, not responded despite WD treatment) Retrospective analysis of 16 WD patients	Mean time at LT: 18.5 years (range 8–40) Mean time of WD treatment: 5.5 years Follow-up: up to 20 years	4 patients had tremor and involuntary movements 2 patients had additional psychiatric symptoms 3 patients had abnormal brain MRI (scales assessment not available)	Resolution of neurological and psychiatric symptoms in all patients Normalization of brain MRI in 1 patient (lack of examination in 3 patients)
Weiss et al. [40]	*N* = 11 (LT indication: 6 with chronic liver disease; 5 with acute LF) Retrospective study of medical records (from 19 patients, analysis was performed in 11 at all time-points, 5 patients deceased, 1 lost to follow-up)	Mean age at LT: 29.3 years (all patients) Mean age of WD diagnosis: 21.6 years (in chronic LF group) Mean time between WD diagnosis and LT: 14.9 years (excluding acute LF) Treatment type and duration not available	12-item neurological scoring system (12–60 pts, where 1 pt is none and 5 pts is severe for each item) 19-item neuropsychological scoring system (0–19 pts, where 1 pt is present) Mean MELD for all 19 patients: 26.4 (not available for neurological patients)	Mean neurological scores at WD diagnosis, at LT and at follow-up improved: Chronic LF: 18.2 pts, 18.4 pts and 13.5 pts Acute LF: 15.6 pts, 18.4 pts and 12.6 pts 1/11 patient worsened, 7 improved, 3 were stable Mean psychiatric scores at WD diagnosis, OLT and at follow-up improved: Chronic LF: 5.8 pts, 6.5 pts and 4.2 pts Acute LF: 4.4 pts, 4.6 pts and 4.2 pts 2/11 patients worsened, 9 were stable
Cheng et al. [42]	*N* = 15 (LT indication: mixed hepatic and neurological presentation) Retrospective analysis of 36 WD patients transplanted due to LF (2 acute LF, 34 chronic decompensated liver disease)	Mean age at LT: 36.9 years (range 29–45) (all patients) From 36 patients, 14 were treated with DPA for 2–9 years Data unavailable for neurological WD patients Mean follow-up: 45.2 months (range 3–88)	Scoring system involving symptoms and functional deficits (0–30 pts, where 0 is worse condition and 30 is no deficits)	Neurological function improved in all survivors Mean results: 16.2 pts pretransplant; 18.2 pts after 6 months; 23.9 pts after 1 year; 26.6 pts after 2 years 6 patients died due to LT complications
Duarte-Rojo et al. [43]	*N* = 2 (LT indication: neurological deterioration) Retrospective analysis of 11 WD patients	Mean age at LT: 23 years DPA, duration not available Follow-up: median 28 months (range 0–80)	Neurological symptoms description Mean MELD: 8.5 (range 6–11)	Remission of all neurological symptoms—patients returned to work
Yoshitoshi et al. [21]	*N* = 4 (LT indication: chronic liver disease) Retrospective analysis of 32 WD patients (21 acute LF, 11 chronic liver disease)	Mean age at LT: 28 years (range 19–40) Mean duration of WD: 16.7 years Patients were treated before with DPA (3) and trientine (1) Mean follow-up: 7 years and 4 months (range 2–15)	Neurological symptoms description	1 patient complete remission of symptoms 1 patient neurological symptoms remained unchanged 2 patients died–1 shortly after LT (bleeding from esophageal varices), 1 due to pneumonia
Martin et al. [46]	*N* = 5 (LT indication: chronic liver disease) Retrospective analysis of 11 WD patients	Mean age at LT: 29.7 years Mean time from WD diagnosis to LT: 8.3 years (range 1–24) All patients treated DPA and/or ZS Mean follow-up: 56.8 months	Neurological symptoms description	4 patients almost completely improved; 1 patient improved Improvement was observed up to 3 years after LT
Pabon et al. [45]	*N* = 2 (LT indication: chronic liver disease) Retrospective study of 13 WD patients	Mean age at LT: 29.7 years Mean time from WD diagnosis to LT: 38 months, treated with DPA or trientine Follow-up: 10 years	Neurological symptoms description	1 patient with persistent neurological symptoms 1 patient improved
Marin et al. [22]	*N* = 4 (LT indication: neurological worsening) Retrospective analysis of 14 WD patients	Age of LT and duration of WD not available All patients were treated with DPA before LT Follow-up: 8 years (range 1–15)	Neurological symptoms description	1 patient died due to acute liver rejection 2 patients were without neurological symptoms 1 patient neurologically improved substantially
Medici et al. [50]	*N* = 9 (LT indication: chronic liver disease) Retrospective analysis of 37 WD patients	Mean age at LT: 27.5 years (range 15–56) (all patients) Mean duration of treatment: 36.2 months Most patients treated with DPA (details not available) Mean follow-up in 33 patients: 64.4 months (range 2–152)	Scoring system for neurological symptoms: rigidity, bradykinesia, ataxia, tremor, dyskinesia, dystonia and walking, eating, talking, daily-living activities (0–30 pts, where 30 is healthy) Psychiatric symptoms described (paranoid psychosis, neurosis, depression, insomnia, drug dependence)	After LT, neurological disability improved in 6/9, regressing completely in 2 cases Neurological deterioration in 3 patients: 1 within 2 months then remained stable 1 developed de novo severe neurological symptoms after LT—pontine myelinolysis and died 1 had neurological impairment after LT but was lost to follow-up (died 10 years later from sepsis) No regression of psychiatric symptoms
Wang et al. [51]	*N* = 11 (LT indication: acute or chronic liver disease) Retrospective analysis of 22 WD patients	Mean age at LT: 13.6 years (range 8–21) (all patients) Mean WD duration: 6.4 years; data on treatment not available Mean follow-up: 18.5 months (range 4–38)	Neurological symptoms description: tremor, bradykinesia, dysarthria, sialorrhea, difficulty in walking (improvement scored in %)	Marked neurological improvement in 10 alive patients between 80–95%
Wang et al. [52]	*N* = 7 (LT indication: acute or chronic liver disease) Retrospective analysis of 18 WD patients	Mean age at LT: 13.5 years (range 6–20) (all patients) Treatment type and duration not available Mean follow-up: 18.2 months (range 2–32)	Neurological symptoms description	7/7 WD patients showed alleviation of a language handicap and dyskinesia
Geissler et al. [53]	*N* = 4 (LT indication: acute or chronic LF) Retrospective study of 6 WD patients	Age at LT and WD diagnosis not provided; 3–24 years’ treatment with DPA (chronic liver disease patients) Follow-up: range 3–7 years	Neurological symptoms description 1 patient had psychiatric symptoms	Neurological recovery in 4 patients 2 patients recovered and returned to work (both complete recovery in 2 patients)
Ronghua et al. [29]	*N* = 15 (LT indication: LF) Retrospective study of 18 WD patients	Age range at LT: 9–23 years Duration of WD not provided DPA for 3 months – 6 years	Neurological symptoms description only (without grading)	Neurological symptoms disappeared in most patients Neurological symptoms persisted in 2/15 patients
Eghtesad et al. [58]	*N* = 17 (LT indication: acute or chronic liver disease) Retrospective analysis of 45 WD patients	Mean age at LT: 22.1 years (range 9–39) Mean age at diagnosis: 15.4 years (range 3–31) Follow-up: range 2–26 years	Neurological symptoms description	Complete neurological improvement was seen in 9/13 survivors 1 other patient improved neurologically 3 did not improved 4 died (2 in group of acute LF and 1 in subacute LF)
Chen et al. [25]	*N* = 7 (LT indication: chronic liver disease) Retrospective analysis of 33 WD patients	Mean age at LT: 21.4 years (range 16–32) (all patients) Treatment type and duration not available Mean follow-up: 62.7 months (range 36–130)	Neurological symptoms description	All 7 patients improved and returned to active lives (5 returned to work, 2 returned to school) 1 died 3 years after LT in a car accident
Bellary et al. [26]	*N* = 9 (LT indication: chronic liver disease) Retrospective analysis of 39 WD patients	Mean age at LT: 23 years (all patients) Mean duration of WD: 10.1 years; data on treatment not available Mean time from diagnosis to LT: 10 years Follow-up: 3–10 years	Neurological symptoms description	2 died within 3 weeks of LT 7 WD patients improved neurologically significantly (90–100% recovery)
Schilsky et al. [62]	*N* = 8 (LT indication: 7 with chronic liver disease, 1 with progressive neurological deterioration) Retrospective study of 55 WD patients	Mean age at LT: 25.5 years Treatment type and duration not available in neurological patients Follow-up: up to 20 years	Neurological symptoms description	4 patients improved neurologically Psychiatric symptoms improved in 1 patient, but gait disturbances remained 3 WD patients died (1 after 6 weeks, 1 after 5 days, 1 after 13 months with AIDS)

AIDS, acquired immunodeficiency syndrome; DPA, d-penicillamine; LF, liver failure; LT, liver transplantation; MELD, Model for End-Stage Liver Disease; MRI, magnetic resonance imaging; mRS, modified Rankin scale; UWDRS, Unified Wilson’s Disease Rating Scale; WD, Wilson’s disease; ZS, zinc salts

Time to LT, time since WD diagnosis and treatment initiation to LT

The cumulative survival rate in studies was 90.4% at the end of follow-up of all included studies. Most studies, case series and case reports reported favorable effects, with major improvement or complete neurological recovery in 215 WD cases (71.2%). In 21 cases (6.9%), there was no difference in neurological status before and after LT. There were 29 deaths (9.6%), 24 cases of de novo neurological deterioration (7.9%; mostly related to immunosuppressive treatment after LT), and 13 cases (4.3%) were lost to follow-up. There was a clear trend toward better results of LT in more recent studies compared with older studies, with older studies suggesting worse outcome in WD patients with neurological presentations [50], which was not confirmed in newer publications (Table 1 and Supplementary Table 1). For example, Lankarani et al. [65] reported improvement of neurological symptoms in almost 67% of transplanted WD patients.

Out of 24 cases and series reports of 32 WD patients, 25 (78%) independent WD patients showed substantial improvement in neurological status and dependence within 4 weeks to 10 years after LT. In four cases (12.5%), there was slight neurological improvement; however, patients were dependent. There was also one deterioration [61] and two deaths [31, 49]. In one of those patients, the patient showed neurological improvement, but then the patient died due to aneurysm rupture [31].

Taking into account the progress in transplantation during the time (surgery techniques as well as immunosuppression and global patient healthcare), we additionally analyzed cases according to when LT was performed: 15 patients underwent LT up to 2000 and 17 had LT after 2000. In the pre-2020 group, we found three cases of slight neurological improvement (patients remained functionally dependent) [31, 32, 60] and one case of neurological deterioration (1/15) [61]. There were no neurological deteriorations described in the group transplanted after 2000. Analyzing deaths after LT, one case was reported before 2000 [31] and one after [49].

The analysis of brain MRI in patients after LT retrieved from hepatological registries was very limited, apart from the study by Poujois et al. [64], which documented a correlation between regression of MRI changes and clinical improvement (Table 1). We found assessment of the brain MRI changes in case and series reports for only 10 out of 32 patients [24, 35, 38, 48, 55–57, 61]. In nine cases, regression of disease was observed [24, 35, 38, 48, 55–57], and in one, there was worsening that corresponded with clinical neurological deterioration [61]. The clear correlations between radiological changes and neurological status were adequately presented only in case reports. However, the methodology (lack of semiquantitative scales, different MRI technology) limited drawing conclusions from these results.

## Discussion

To our best knowledge, this is the first systematic review of the literature that presents the effectiveness of LT in the treatment of neurological manifestations of WD.

Based on international recommendations, LT is recommended only for WD patients with acute liver failure or in decompensated liver cirrhosis [1–5]. All patients should also fulfill modified Nazer index as a prognostic factor for survival in WD patients and LT [1–5]. According to international registries, about 1.5% of LT are performed due to WD. Based on WD registries, around 5% of WD patients are treated with LT, mostly due to acute liver failure [1–3].

The first experiences with LT in WD were reported in 1963 when DuBois et al. [20] performed the first-ever LT in WD patient with acute liver failure. The first report of neurological outcome after LT was published in 1973 by Groth et al. [16]. They described a 14-year-old boy with WD, hepatic symptoms, and progressive neurological deterioration (dystonia, choreoathetosis, and dysarthria), despite treatment with d-penicillamine since the age of 11 years. During the first 12 months after LT, neurological symptoms improved and completely resolved within 4 years [16, 17]. Then in 1983, Zitelli et al. [18] described successful LT in a 13-year-old WD patient, with subsequent resolution of neurological symptoms 15 months after transplantation. These cases, as well as a lack of treatment in severe neurological WD patients, led to several studies of LT in patients with neurological symptoms over the next 40 years.

After initial reports of LT in patients with unresponsive neurological WD, Cheng et al. [42] found that WD patients with neurological symptoms had worse prognosis for survival compared with WD patients without neurological presentation (80% vs. 100% after 1 year, 60% vs. 100% after 3 years and 60% vs. 89.5% after 5 years). They documented that the presence of neurological symptoms (especially severe) was a statistically important factor that negatively affected survival after LT (6/15 patients died over the 5-year follow-up). However, it should be noted that the neurological improvement of some degree was observed in all survived WD patients [42].

Similar observations were provided by Medici et al. [50] who reported post-LT overall survival rates of 89% after 1 year, 82.9% after 3 years, 75.6% after 5 years, and 58.8% after 10 years. Lower survival rates were associated with neuropsychiatric WD presentation (as the authors neurological and psychiatric patients analyzed collectively), with mean survival of 135 months with the hepatic form vs. 79 months in patients with additional neuropsychiatric presentation. Importantly, in 6/7 (85%) of WD patients with neurological symptoms who survived and were followed up (1 was lost to follow-up), significant improvement of neurological symptoms was observed, with complete resolution of symptoms in two cases [50].

More recent studies involving larger groups of WD patients did not confirm previous observations suggesting worse prognosis in neurological WD patients. Ferrarese et al. [27] and Lankarani et al. [65] did not observe an adverse influence of neurological presentation on survival rates in WD patients after LT. Moreover, these studies documented better long-term general survival rates in transplanted WD patients: 88% after 1 year and 83% after 5 years [27]; 86% after 1 year and 82% after 5 years [65]. However, contrary to previous reports, in the registry described by Ferrarese et al. [27], no significant improvement of neurological WD patients (*n* = 10) after LT was observed. The Iranian study by Lankarani et al. [65] did not confirm that neurological symptoms affected survival, but found improvement of neurological symptoms in almost 67% of transplanted WD patients. The disparity between older and more recent studies may be due to improvements in transplantology techniques as well as more accurate qualification for LT over time, which has contributed to better results in neurological WD patients.

A recent retrospective analysis of a French registry of LT in WD presented very similar findings to Lankarani et al. [65] and Cheng et al. [42], with general survival rates of 89% after 1 year and 87% after 5 years [19]. However, in multivariate analysis, there was a strong statistical trend (*p* = 0.06) toward poorer survival in patients with neurological symptoms [19]. The authors also documented major neurological improvement in 3/6 cases transplanted due to neurological indications, meaning that all WD patients who survived had neurological improvement. The other more recent study performed by Poujois et al. [64] showed better survival results in neurological WD patients (88.8% after 1 year and 72.2% after 3 and 5 years), which are very similar to general populations undergoing LT (89% after 1 year in a French registry). These results clearly demonstrate that each new study with LT in WD patients show better results and survival, also in neurological WD patients, which allows us to be optimistic about the future of LT in WD patients with neurological presentation not responding to anti-copper treatment. Of note, Poujois et al., first used the objective Unified Wilson’s Disease Rating Scale (UWDRS) and documented neurological improvement in 8/18 (44%) of severe neurological patients (bedridden, not responsive to treatment) and in 8/14 (67%) WD patients who survived. In our opinion, these results are the first to suggest that LT could be an effective treatment in WD patients with severe neurological presentation. The question is when to decide about LT? Sufficient time must be given for anti-copper treatment to work, but it is important not to act too late, when WD progression has caused irreversible brain damage as well as neurological symptoms with contractures and secondary chronic infections.

Discussing the results of case reports, it is worth mentioning that the presented results of LT in WD documented improvement in results in last two decades. Patients who were transplanted more than 20 years ago less frequently achieved significant neurological improvement. These data are consistent with world-wide findings indicating markedly improved post-LT outcomes over the last 30 years [75]. For example, in France, rates of 1-year post-transplant survival increased from 78% in 1990–1994 to 85% from 2005–2007 [75]. These results were achieved due to several factors, including better selection of LT candidates, donor selections, organ procurement, surgical techniques, post-operative care, immunosuppression, as well as general patient management [75].

There is a lack of large studies prospectively analyzing the impact of LT on improvement of symptoms and outcome of WD patients with neurological presentation [76]. Data from retrospective studies, case and series reports suggest neurological improvement in 71% of patients and neurological improvement or stabilization in 78% of cases. It should be mentioned that WD patients who underwent LT in the reviewed publications had mostly severe neurological deficits and/or liver failure. Although results from the retrospective studies and case reports showed that neurological symptoms are risk factors for worse survival after LT, they were not confirmed in more recent studies [27, 64, 65].

Summarizing, the presented results suggest that LT is a promising method of WD treatment, particularly if patients have not responded to pharmacological de-coppering treatment.

## Limitations

Our literature review has some limitations. Firstly, all 48 papers were retrospective. All studies used various endpoints and methods for their evaluation. Only more recent studies used neurological WD scales (UWDRS) [64] or WD scales developed by Medici et al. [50]. In older studies, only neurological symptoms were described. Further, the studied groups were not homogenous; most of the patients from registries were transplanted due to hepatic indications (mild neurological symptoms), while others, such as the Poujois et al., study and most case reports, were transplanted due to severe neurological deficits. Apart from a few case reports, only Poujois et al. [64] used brain MRI scales for quantitative assessment of the brain. Also, in most of the articles, there was a lack of hepatic assessment according to detailed scales that are useful for LT qualification, such as Model for End-Stage Liver Disease (MELD) or Nazer score, which would be helpful to verify the hepatological indications for LT in neurological patients.

Regarding analyzing the impact of LT on neurological WD symptoms, some of the reported neurological (or neuropsychological/psychiatric) improvements could be due to resolution of hepatic encephalopathy (even subclinically in the case of neuropsychological functioning). Further, we have mentioned, but not analyzed, psychiatric improvement after LT, due to too little data [76, 77]. However, due to the high frequency and clinical significance of psychiatric and cognitive disturbances in WD, this issue should be analyzed separately.

In some of the reviewed studies, chelators or zinc salts were used in addition to LT to remove copper from organs [42, 65]. However, recommendations for such combined treatment are not available [1–3].

Finally, there is increasing recognition of the importance of early detection of WD as it has been shown that presymptomatic individuals, when properly treated with anti-copper agents, do not develop clinical symptoms, including neurological symptoms [1–4, 77]. According to WD pathophysiology, neurological symptoms usually occur a few years after hepatic symptoms and many years after the presymptomatic stage of the disease [1–4]. We are currently seeing substantial improvements in WD diagnosis [77–81], including better knowledge of WD by physicians, genetic testing implementation [78–80], and more common family WD screening [4, 80]. There are also suggestions to include neonatal screening for WD using DNA or quantitative assessment of ATP7B protein in dried blood spots [81]. Additionally, new WD treatment modalities are currently under investigation [82] and they may decrease the number of WD patients with neurological presentation [83, 84]. It gives hope that in the near future, discussions surrounding how to treat severely impaired neurological WD patients will be less necessary.

## Conclusions

Currently available data encourage the use of this treatment option, especially in severe neurological patients not responding to anti-copper treatment; however, it is still uncertain which patients with neurological impairment benefit most from LT and when is the optimal timing for LT [77].

Further studies conducted on large cohorts, using neurological and radiological scales as well as potential biomarkers of neurological injury, are needed to establish the role of LT in the treatment of WD with neurological symptoms.

## Supplementary Information

Below is the link to the electronic supplementary material.

Supplementary file1 (DOC 84 kb)
