# Supplementary material for: Liver transplantation as a treatment for Wilson’s disease with neurological presentation: a systematic literature review
Source: Acta Neurol Belg. 2022 Jan 26;122(2):505–18. doi: 10.1007/s13760-022-01872-w (PMC8986686; doi:10.1007/s13760-022-01872-w)
Supplement: Supplementary file 1 — Supplementary file1 (DOC 84 kb) [file 13760_2022_1872_MOESM1_ESM.doc]

**Supplementary material**

**Supplementary table 1**. Summary of case and series reports of WD patients with neurological symptoms who underwent liver transplantation

| **Authors** | **Details of the patient population and study** | **Information on age at LT; duration and type of prior treatment; duration of follow up (if available)** | **Details of the neurological assessment and MELD score before LT (if available)** | **Results** |
| --- | --- | --- | --- | --- |
| Walker et al. (2018)  [34] | N = 1 (LT indication: hepatic carcinoma)  Case report | Age at LT: 45 years; 31 years’ treatment with DPA and/or ZS, with exacerbation of neurological symptoms 2 years before LT  Follow-up: 18 months | Neurological symptoms description: bradyphrenia, immobilized with severe parkinsonism | - Gradual neurological recovery from 9–18 months after LT - Mild action tremor and slight rigidity persisted, functionally independent |
| Haberal et al. (2017) [71] | N = 1 (LT indication: chronic liver disease)  Case report | Age at LT: 36 years; chelation treatment (unknown duration and type of drug) Follow-up: 7 months | Neurological symptoms description: tremor, walking disturbances, dysarthria | - Completely regression of neurological symptoms |
| Laurencin et al. (2017) [35] | N = 4 (LT indication: 2 had neurological worsening; 2 had LF)  Series reports | Age at LT: 17, 19 15 and 23 years; treatment from 1 month to 2 years before LT  Follow-up: 21 months to 4 years | mRS, UWDRS, brain MRI  MELD:   - 8 points - 6 points - 21 points - 40 points | - Neurological improvement in 4 patients - 1 patient with neurological worsening: mRS from 4 to 3 and UWDRS from 79 to 45 - 1 patient with neurological worsening: mRS from 4 to 2 and UWDRS from 74 to 28 - 1 patient with hepatic worsening: mRS from 2 to 0 and UWDRS stable at 1 - 1 patient with hepatic worsening: mRS from 1 to 0 (UWDRS not available) - Partial regression of brain MRI changes in 3 cases, stabilization in 1 case |
| Sutariyaa et al. (2015) [37] | N = 1 (LT indication: neurological deterioration)  Case report | Age at LT: 14 years; 2 years’ treatment with DPA and ZS (stopped by patients)  Follow-up: 1 year | Neurological symptoms description: bedridden, severe rigidity, dystonia, tremor and bradykinesia Psychiatric symptoms description: irritability, mood disturbances and behavioral changes | - 6 months after LT, the patient could walk without support, with normal gait and speech - 1 year after LT, the patient stopped taking immunosuppressive drugs, experienced graft rejection and died |
| Dou et al. (2014) [39] | N = 1 (LT indication: chronic liver disease)  Case report | Age at LT: 12 years; 1 years’ treatment with DPA and ZS  Follow-up: 5 years | Neurological symptoms description: tremor-rigidity syndrome, dysarthria | - Patient described as “free of neurological symptoms” |
| Mocchegiani et al. (2014) [38] | N = 1 (LT indication: neurological deterioration)  Case report | Age at LT: 19 years; 9 years’ treatment with DPA and ZS Follow-up: 4 years | Scoring system for neurological symptoms: rigidity, bradykinesia, ataxia, tremor, dyskinesia, dystonia and walking, eating, talking, daily-living activities (0–30 pts, where 30 pts is healthy)  Routine brain MRI | - Marked neurological improvement from 8 pts to 28 pts - Brain MRI: - T2 sequences initially showed hyperintensive signals in basal ganglia, thalami, mesencephalon (giant panda) - Signals had disappeared 4 years later |
| Park et al. (2008) [44] | N = 1 (LT indication: chronic liver disease)  Case report | Age at LT: 36 years; 14 years’ treatment with DPA  Follow-up: 26 months | Neurological symptoms description: intention tremor  MELD: 17 points | - Neurological symptoms completely recovery |
| Suess et al. (2007) [48] | N = 1 (LT indication: neurological deterioration)  Case report | Age at LT: 33 years; 1 years’ treatment with DPA  Follow-up: 3 years | Neurological symptoms described and video-taped  Routine brain MRI routine | - Improved neurological symptoms were observed, with the patients described as “nearly free of neurological symptoms apart from mild dysarthria and slight tremor of right hand” - Improvement in brain MRI, with almost complete resolution of bilateral changes in thalami in T2 sequences |
| Senzolo et al. (2006) [49] | N = 2 (LT indication: chronic liver disease)  Case reports homozygotic twins | Patient 1: age at LT: 32 years; 2 years’ treatment with ZS  Patient 2: age at LT: 34 years; 10 years’ treatment with ZS  Follow-up: 10 years | Neurological symptoms description  Patient 2 also had psychiatric symptoms | - Patient 1: Complete neurological recovery - Patient 2: Died |
| Suzuki et al. (2003) [54] | N = 1 (LT indication: neurological deterioration)  Case report | Age at LT: 17 years; 10 months’ treatment with DPA and ZS  Follow-up: 12 months | Neurological symptoms description: dystonia, dysarthria | - Almost normalization of neurological examination (residual dysarthria persisted) |
| Hermann et al. (2002) [55] | N = 1 (LT indication: neurological deterioration)  Case report | Age at LT: 21 years; 2 years’ treatment with DPA  Follow-up: 3 years | Neurological symptoms evaluated based on presence and severity: dysarthria, tremor, ataxia, motor disorder, dysdiadochokinesia, bradykinesia, facial dystonia, swallowing disorders, everyday activities (0–24 pts) Routine brain MRI | - Gradually improvement of neurological symptoms in 3 years (from 21/24 to 8/24) - Improvement in daily activities (from initially dependent to not dependent) - Reduction of brain MRI changes (discrete hypodense signals of basal ganglia) after 3 years |
| Stracciari et al. (2000) [57] | N = 1 (LT indication: neurological deterioration)  Case report | Age at LT: 44 years; 6 months’ treatment with DPA  Follow-up: 18 months | Neurological examination severity index: tremor, dystonia, ataxia, dysarthria and total (where 0 is absent and 4 is extremely severe; 0–16 pts)  Multiple neuropsychological scales  Brain MRI | - Resolution of neurological symptoms in 9 months (from 11/16 to 0/16) - Improvement in neuropsychological tests (Wechsler Adult Intelligence Scale; Rey Figure copy, and several others) - Resolution of brain changes (attenuation of the basal ganglia signal) after 18 months |
| Wu et al (2000) [56] | N = 1 (LT indication: chronic LF not responded to chelators, with gradual neurological deterioration)  Case report | Age at LT: 35 years; 19 months’ treatment with DPA  Follow-up: 16 months | Neurological symptoms evaluated based on presence and severity: dysarthria, tremor, ataxia, masked face, dystonia, rigidity, handwriting (scored from + to +++)  Routine brain MRI | - Resolution of neurological symptoms (from 18+ to 1+) with persistence of slight dysarthria at 16 month after LT - Resolution of brain MRI changes (changes in both thalami and red nuclei) after 16 months |
| Bax et al. (1998) [59] | N = 1 (LT indication: neurological deterioration)  Case report | Age at LT: 14 years; 3 months’ treatment with DPA and ZS, after 3 months of ZS  Follow-up: 1 year | Neurological scoring system (10 parameters scored 0–3 pts each) | - Improvement in neurological score from 19 to 5 in 9 weeks, then reduced to 2 after 1 year (with only elevated muscle tone in left upper extremity) |
| Kassam et al. (1998) [32] | N = 1 (LT indication: chronic liver disease) Case report | Age at LT: 22 years; 12 months’ treatment with DPA  Follow-up: 43 months | Neurological symptoms description: dystonia, dysarthria to anarthria, dysphagia, progression of neurological symptoms until immobilization | - Neurological improvement: could move all four limbs and transfer from bed to chair 3 months after LT - Further improvement in mobility after 26 months - Suicide 43 months after LT |
| Lui et al. (1998) [24] | N = 1 (LT indication: chronic liver disease)  Case report | Age at LT: 17 years; 4 years’ treatment with DPA  Follow-up: 4-years | Neurological symptoms description  Brain MRI analysis | - Significant neurological improvement - Reversal of brain MRI pathology |
| Schumacher et al. (1997) [60] | N = 4 (LT indication: intractable neurological impairment)  Series reports | Mean age at LT: 27 years (range 15–34) Follow-up: up to 5.5 years | Description of neurological symptoms: dysarthria, tremor, ataxia, sialorrhea, dystonia, handwriting difficulties | - All patients revealed neurological improvement 4–6 weeks after LT - Long-term follow-up: - 1 patient completely recovered - 2 noticeably improved (residual symptoms persisted) - 1 only slight improvement |
| Tison et al. (1996) [69] | N = 1 (LT indication: neurological worsening)  Case report | Age at LT: 19 years; 7 years’ treatment with DPA, worsening after treatment cessation, lack of improvement after 1 year of treatment re-introduction | Neurological symptoms description: disabling postural tremor, dysarthria, and bradykinesia  Brain MRI | - Major neurological improvement, slight dysarthria remained - Graft rejections, hepatic artery thrombosis (third transplantation successful) |
| Guarino et al. (1995) [61] | N = 1 (LT indication: chronic liver disease) Case report | Age at LT: 27 years; 3 months’ treatment with DPA  Follow-up: 19 months | Neurological symptoms description: dysarthria, gait ataxia, bradykinesia  Neuropsychological tests: WAIS, Rawen’s matrices, etc.  Routine brain MRI | - Neurological worsening with tetraparesis 19 months after LT - Worsening in neuropsychological tests - Worsening in brain MRI: initial changes in caudate and putamen persisted, additional central pontine and extrapontine myelinolysis (cerebellar hemispheres) occurred |
| Mason et al (1993) [31] | N = 1 (LT indication: progressive neurological deterioration)  Case report | Age at LT: 25 years; 5 years’ treatment with DPA and BAL  Follow-up: 4 weeks (until death) | Neurological symptoms description: dystonia, dysarthria to anarthria, depression | - Neurological improvement was observed - The patient started to talk 3 weeks after LT, could defecate without help, drooling and dysphagia diminished - Sudden death 4 weeks after LT (rupture of splenic aneurysm) |
| Hefter et al. (1991) [70] | N = 1 (LT indication: chronic LF with portal hypertension)  Series report of 2 WD cases | Age at LT: 19 years; 4 months’ treatment with DPA  Follow-up: 6 years | Neurological symptoms description: progressive lethargy, dysarthria, bradykinesia, hypomimia, ptosis, swallowing problems | - Recovery, with marginal bradykinesia and dysarthria |
| Polson et al. (1987) [30] | N = 2 (LT indication: chronic liver disease)  Case reports | 1 patient: 30 years at LT; 14 months’ treatment with DPA  1 patient: 27 years at LT; 7 months’ treatment with DPA, later trientine  Follow-up: 8 months | Neurological symptoms description:  1 patient: dysarthria, dysphagia, akinesia and rigidity, required constant nursing care  1 patient: dysarthria, akinesia, tremor and psychological impairment | - 1 patient had no neurological symptoms after 8 months - 1 patient improved substantially, with mild dysarthria and tremor and steady gait 3 months after LT - Further improvement was seen after 5 months (behavioral symptoms remained) |
| Zitteli et al. (1983) [18] | N = 1 (LT indication: subacute LF)  Case report | Age at LT: 13 years; treatment with DPA  Follow-up: 15 months | Neurological symptoms description: ataxia and cogwheel rigidity | - No neurological symptoms after 15 months |
| Groth et al. (1973) [16] | N = 1 (LT indication: neurological deterioration)  Case report | Age at LT: 14 years; 3 years’ treatment with DPA  Follow-up: 50 months | Neurological symptoms description: dysarthria, dystonia, choreoathetosis | - No neurological symptoms after 50 months (improvement noticed after 18 months) |

BAL = British anti-Lewisite; DPA = d-penicillamine; LF = liver failure; LT = liver transplantation; MELD = Model for End-Stage Liver Disease; MRI = magnetic resonance imaging; mRS = modified Rankin scale; UWDRS = Unified Wilson’s Disease Rating Scale; WAIS = Wechsler Adult Intelligence Scale; WD = Wilson’s disease; ZS = zinc salts.
